# Thermal rearrangement of *tert*-butylsulfinamide

**DOI:** 10.3762/bjoc.7.2

**Published:** 2011-01-04

**Authors:** Veera Reddy Arava, Laxminarasimhulu Gorentla, Pramod Kumar Dubey

**Affiliations:** 1 R&D Laboratories, SUVEN Life Sciences Ltd, #18, Phase III, Jeedimetla, Hyderabad – 500 055, India; 2 Department of Chemistry, J.N.T. University, Hyderabad – 500 072, India

**Keywords:** chlorinated solvents, *N*-(*tert*-butylthio)-*tert*-butylsulfonamide, *tert*-butylsulfinamide, thermal rearrangement

## Abstract

*tert*-Butylsulfinamides are unstable above room temperature, and in chlorinated solvents they undergo rearrangement to form the more stable *N*-(*tert*-butylthio)-*tert*-butylsulfonamide.

## Introduction

**Figure 1:**
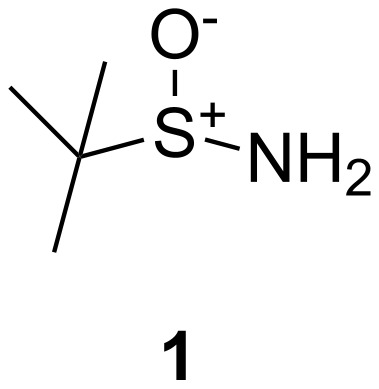
*tert*-Butylsulfinamide.

Over the past decade, an ever increasing number of methods based upon the chiral amine reagent *tert*-butylsulfinamide (**1**) (Figure 1) has become one of the most extensively used synthetic approaches for both the production and discovery of drug candidates [1]. In particular, the *tert*-butylsulfinyl group showed high levels of asymmetric induction in many processes. The importance of these reagents (*R* and *S*) is evident by the number of manufacturers (>75) and by the number of publications (>400).

## Results and Discussion

**Scheme 1:**
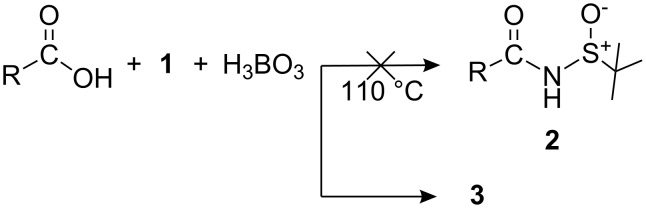
Synthesis of acid amide.

During our studies [2] on the industrial utilization of these two reagents (*R* and *S*), we found an interesting observation, i.e., when sulfinamide **1** was reacted with an organic acid in the presence of boric acid [3] to obtain an amide **2** (Scheme 1), no amide was obtained, which was confirmed by IR and NMR data, and the product was also devoid of an acid residue. The product appeared to be derived only from the reagent **1**.

**Figure 2:**
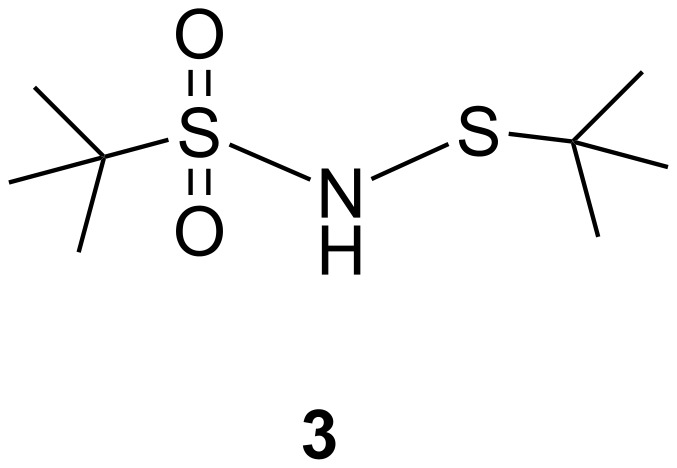
*N*-(*tert*-butylthio)-*tert*-butylsulfonamide.

**Scheme 2:**
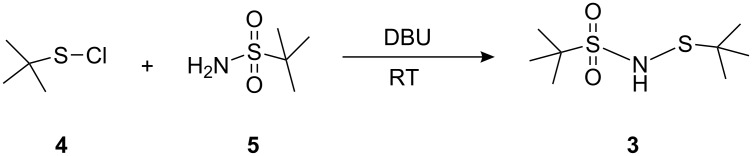
Chemical synthesis of **3**.

**Figure 3:**
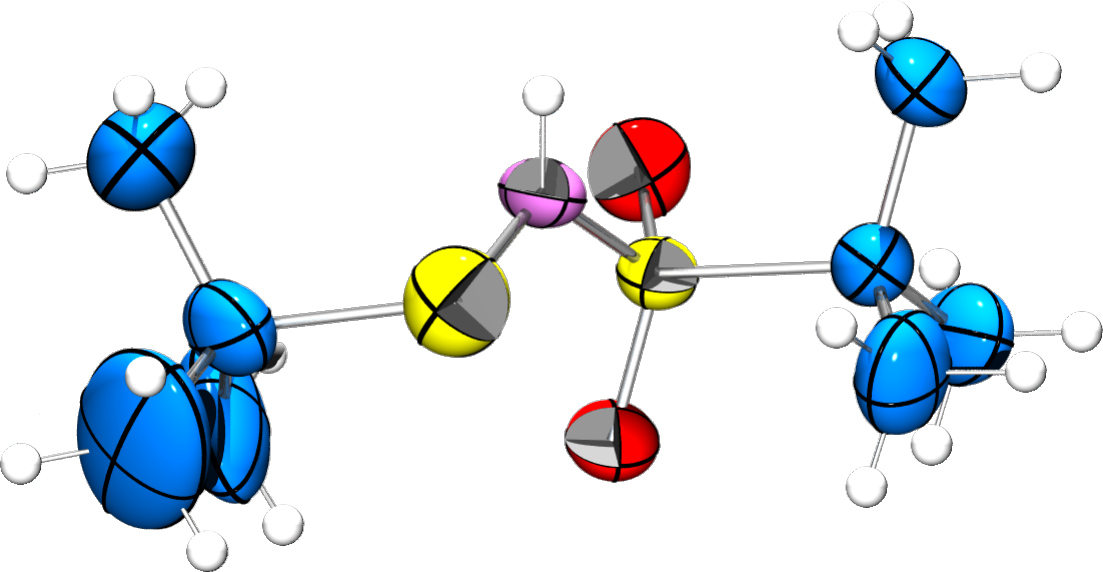
ORTEP diagram of **3**.

**Scheme 3:**
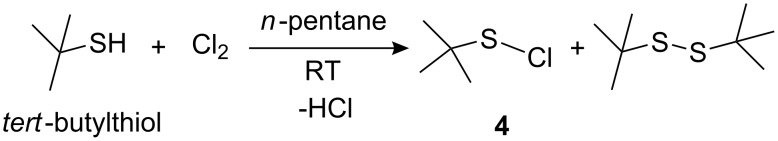
Synthesis of *tert*-butylsulfanyl chloride.

**Scheme 4:**
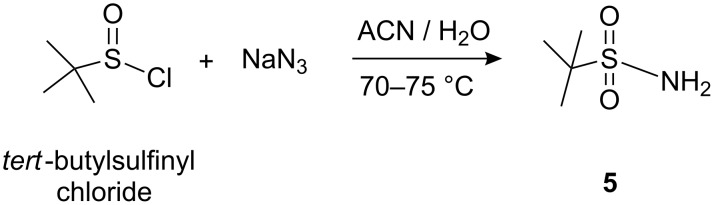
Synthesis of *tert*-butylsulfonamide.

From the spectral data, structure **3** was assigned to the product shown in Figure 2. The structure of **3** was confirmed by chemical synthesis (Scheme 2) and finally by XRD [4] (Figure 3). Both *tert*-butylsulfanyl chloride **4** (Scheme 3) and *tert*-butylsulfonamide **5** (Scheme 4) were prepared by known procedures [5–6].

**Scheme 5:**
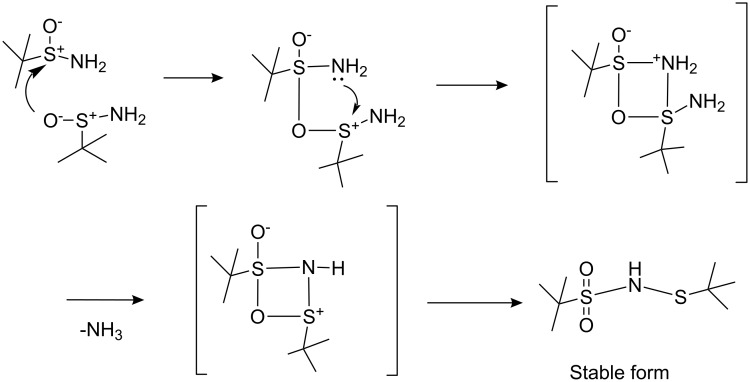
Proposed mechanism for rearrangement.

A possible mechanism for the proposed rearrangement is shown in Scheme 5.

First it is assumed that the product is formed only by the degradation of reagent **1.** Further experiments, in the presence of acids (entries 7 to 13, Table 1) or the absence of acids (entries 1 to 6, Table 1), under sonication (entries 14 to 18) and with microwave irradiation (entry 26), confirmed the assumption that the reagent is thermally unstable. Also the rearrangement is likely not to proceed by a homolytic fission (radical) mechanism, because the rate of reaction is not affected either by benzoyl peroxide, by TEMPO a radical initiator (entries 22 and 23) or by a radical inhibitor 2,6-di-*tert*-butylphenol (entry 24).

**Table 1:** Screening of rearrangement conditions.

**Entry**	**Reaction conditions**					**Yield** (%)^a^
	Starting material	Reagent / condition type	Solvent	*T* (°C)	Time (h)	

1	(*R*)-isomer	-	-	110	3	27
2	(*R*)-isomer	-	toluene	110	48	70
3	(*R*)-isomer	-	*o*-xylene	140	48	64
4	(*R*)-isomer	-	ethylene dichloride	80	72	40 + 20^c^
5	(*R*)-isomer	-	CHCl_3_	65	72	25
6	(*R*)-isomer	-	toluene	110	48	70
7	(*R*)-isomer	boric acid (1.0 equiv)	toluene (u/N_2_)	110	24	65
8	(*R*)-isomer	boric acid (1.0 equiv)	toluene	110	24	65
9	(*R*)-isomer	boric acid (0.5 equiv)	toluene	110	48	60
10	(*R*)-isomer	MeSO_3_H	toluene	110	1	27
11	(*R*)-isomer	tartaric acid	toluene	110	24	38
12	(*R*)-isomer	citric acid	toluene	110	24	16
13	(*R*)-isomer	*p*-TSA	toluene	110	24	38
14	(*R*)-isomer	sonication	CHCl_3_	RT	2	34^b^
15	(*R*)-isomer	sonication	DMF	RT	1	2^b^
16	(*R*)-isomer	sonication	ethyl acetate	RT	1	5^b^
17	(*R*)-isomer	sonication + boric acid	CHCl_3_	RT	1	16^b^
18	(*R*)-isomer	sonication + *p*-TSA	CHCl_3_	RT	0.5	23^b^
19	(*R*)-isomer	-	toluene	RT	144	3
20	(*R*)-isomer	-	CHCl_3_	RT	144	21^b^
21	(*R*)-isomer	-	MeOH	RT	144	3^b^
22	(*R*)-isomer	benzoyl peroxide	toluene	110	48	60
23	(*R*)-isomer	TEMPO	toluene	RT - 110	40	9^b^
24	(*R*)-isomer	2,6-di-*tert*-butylphenol	toluene	110	24	75
25	(*S*)-isomer	-	toluene	110	48	70
26	(*R*)-isomer	MW 150 Watt	DMF	150	0.5	70

^a^Isolated yield, ^b^% conversion in HPLC, ^c^recovered starting material with SOR +5°

When the reagent **1** alone was subjected to thermal rearrangement (entry 1), complete consumption of starting material was observed. Only 27% product was isolated and 73% of the material was lost by vaporitation. When the reaction was carried out in the presence of solvents such as toluene (entries 2, 6 and 24), *o*-xylene (entry 3), or solvents with reagents such as boric acid (entries 7, 8 and 9), methanesulphonic acid (entry 10), *p*-TSA (entry 13), benzoyl peroxide (entry 22), 2,6-di-*tert*-butylphenol (entry 23) or with microwave irradiation (entry 25), complete consumption of starting material was observed. In other cases (entries 4, 5, 11 and 12), 10 to 30% of the starting material was recovered without racemization.

## Conclusion

We found that both (*R* and *S*) *tert*-butanesulfinamides are unstable above room temperature and in chlorinated solvents.

## Experimental

See Supporting Information File 1 for full experimental data.

## Supporting Information

File 1 Full experimental data.
