# Supplementary material for: Thermal rearrangement of tert-butylsulfinamide
Source: Beilstein J Org Chem. 2011 Jan 4;7:9–12. doi: 10.3762/bjoc.7.2 (PMC3028921; doi:10.3762/bjoc.7.2)
Supplement: File 1 — Full experimental data. [file Beilstein_J_Org_Chem-07-09-s001.pdf]

Supporting Information

for

Thermal rearrangement of *tert*-butylsulfinamide

Veera Reddy Arava[*1], Laxminarasimhulu Gorentla[1], Pramod Kumar Dubey[2]

[1]R&D Laboratories, SUVEN Life Sciences Ltd, #18, Phase III, Jeedimetla,

Hyderabad – 500 055, India.

[2]Department of Chemistry, J.N.T University,Hyderabad 500 072, India

Page No

Experimental Section S2

Chemical synthesis of *N*-(*tert*-butylthio)-*tert*-butylsulfonamide (3)

Rearrangement of *tert*-butanesulfinamide S3

General Procedure

Chemical synthesis of *N*-(*tert*-butylthio)-*tert*-butylsulfonamide (3)

tert-Butylsulfonamide (2.4 g, 0.017 mol) was dissolved in 12.0 mL ethyl acetate and DBU (5.3 g, 0.034mol) added at ambient temperature. *tert*-Butylsulfenyl chloride (~2.2 g, 0.017 mol, based on app. 80% yield) in *n*-pentane (10.0 mL) was added dropwise at 25–30 °C over a period of 10 minutes. After complete addition, the reaction mixture (pale yellow sticky mass) was stirred for 1 hour (progress of the reaction was monitored on precoated silica gel plates and visualized with iodine vapors). Water (10.0 mL) was added and the layers were separated. The aqueous layer was extracted once with ethyl acetate (10.0 mL), the combined organic layers were washed twice with water (10.0 mL) and dried over anhydrous Na_2SO_4. Evaporation of the solvent under vacuum resulted in a residue, which was mixed with ether (10.0 mL) and *n*-hexane (20.0 mL) and the crystals were filtered and washed with ether (1.5 mL). The product was purified by column chromatography with 4% ethyl acetate:*n*-hexane as eluent to afford 1.18 g of **3** as white crystals.

Yield: 30%; mp: 159.8–161.8 °C; HPLC Purity: 99%;

IR (cm^{-1}, KBr): 3238 (sharp peak of -NH), 1364 (sharp peak of asym $–SO_2$), 1298 (sharp peak of sym $-SO_2$), 889 (sharp peak of S-N).

^{1}H NMR (TMS/$CDCl_3$): δ = 1.32 (s, 9H, $C(CH_3)_3$, 1.43 (s, 9H, $C(CH_3)_3$, 5.18 (s, 1H, -NH).

^{13}C NMR (TMS/$CDCl_3$): δ = 24.62, 28.00, 48.46 and 61.14.

ESI-MS (-Ve mode): m/z (%) = 224.3 [M-1].

Rearrangement of *tert*-butylsulfinamide

tert-Butylsulfinamide (10.0 g, 0.08 mol) was dissolved in toluene (100.0 mL) at room temperature. The resulting clear colorless solution was heated under reflux and stirred for 48 hours (progress of the reaction was monitored by TLC and the spots were visualized with iodine vapors). After the completion of the reaction, as determined by TLC, the solution was cooled to rt and washed twice with water (50.0 mL). The toluene layer was dried over anhydrous Na_2SO_4 (10.0 g) and concentrated under vacuum. The obtained solid was slurried in *n*-hexane (50.0 mL) at rt and filtered to obtain a white crystalline solid (94% purity), which was recrystallized from ethyl acetate.

Yield: 70%; mp: 162–164 °C; HPLC Purity: 99.5%; $[\alpha]_D^{20}$ (c = 1.0, $CHCl_3$): 0°.

HPLC Conditions

Column: Ymc pack C8 250X4.6mm 5μm (SRC-598); Eluent: Mobile phase A: Water pH adjusted to 2.5 with orthophosphoric acid; Mobile phase B: Acetonitrile; Flow rate: 1.0 mL/min; Detector: UV (210 nm).
